# Strengthening the Referral System through Social Capital: A Qualitative Inquiry in Ghana

**DOI:** 10.3390/healthcare5040080

**Published:** 2017-10-25

**Authors:** Padmore Adusei Amoah, David R. Phillips

**Affiliations:** 1 Division of Graduate Studies and Asia Pacific Institute of Ageing Studies, Lingnan University, 8 Castle Peak Rd, Hong Kong, China; 2 Department of Sociology and Social Policy, Lingnan University, Hong Kong, China; phillips@LN.edu.hk

**Keywords:** health policy, healthcare, referral policy and pathways, social capital, Ghana

## Abstract

The referral system in health care has been noted as very influential in determining which services are accessed and when. Nonetheless, existing studies have relied on specific measurable factors relating to health personnel, transportation and communication infrastructure, and finance to explain the challenges facing the referral policy in developing countries. While this is understandable, the role of social capital remains mostly uncharted even though it is implicit in the well-known lay referral system. Using various facets of the social capital concept, this paper empirically examines how the resources embedded in both structural and cognitive aspects of social relationships influence knowledge of, and adherence to, referral policy. This study is based on semi-structured in-depth interviews conducted with 79 adults in the Ashanti Region of Ghana in 2015. Of the 79 participants, 28 lived in urban areas and 51 in rural localities. Eight health personnel and eight community leaders also contributed to the study. Additionally, six focus group discussions were held. The findings indicated that both cognitive and structural forms of social capital considerably underpinned the ability and willingness of people to adhere to the referral process. Moreover, the role of social capital was double-barrelled. It contributed in a significant way to encouraging or dissuading potential patients from rightly embracing the policy. In addition, precepts of social capital reinforced both positive and adverse effects of the other determinants of the policy such as finance and transportation. However, the magnitude of such impact was linked to how ‘resourceful’ and ‘trustworthy’ one’s available social acquaintances were. The paper suggests that a cautious engagement with social capital will make it a potentially powerful tool for understanding the gaps in and improving the effectiveness of referral policy.

## 1. Background

**Figure 1:**
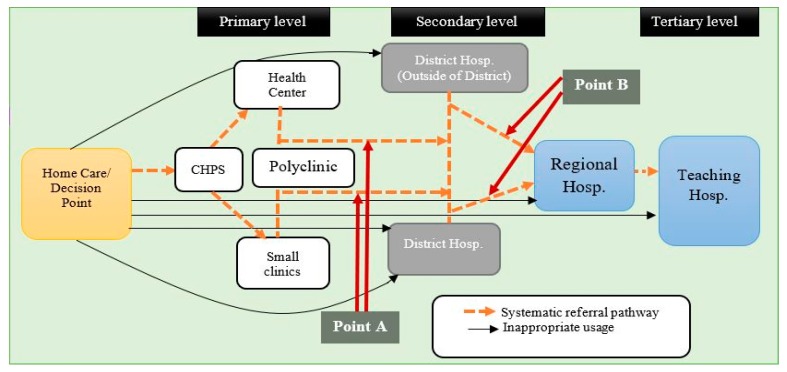
The referral pathway in Ghana. Source: Author’s elaboration, Adapted from BASICS II and GHS [9].

Ghana has one of the best developed health systems in sub-Saharan Africa in terms of availability of health facilities and personnel [1,2]. The country operates a pluralistic health system including the conventional and unconventional sectors as well as the popular sector adequately [3,4]. Nevertheless, the Ghanaian health system remains bedevilled by numerous challenges including a weak referral system [5,6,7]. A formal referral system requires patients to first access primary care and then be referred to an appropriate higher level when the need arises [7,8]. Qualified health personnel are expected to recommend, advise and monitor the process [2,7,9]. In Ghana, patients/clients in the conventional health system are expected to access services from primary services incrementally (e.g., the Community-based Health Planning Services, CHPS, and health centres), through secondary facilities (e.g., district hospitals) and if required to the highest services (regional and tertiary hospitals). The broken lines in Figure 1 demonstrate these idealised steps. The referral system is held to ensure cost-effective and optimal use of health services to the advantage of both patients and the health system [8]. 

However, from the supply side of the health system, the standard procedure for referral is often either absent or hardly put to use in many health facilities in Ghana [5,6]. Some studies in Ghana [10] and elsewhere in sub-Saharan Africa (Uganda, Zimbabwe) [11,12] indicate that disregard for the referral system is associated with inadequate emergency transportation services and telecommunication services, and inadequate health personnel to enable the processing of patients. Moreover, health promotion and outreach programmes have barely touched on the referral policy [2,5,9]. The policy itself concentrates almost exclusively on the supply side of the health system. In Ghana and other places, for practical purposes, patients/clients often make secondary, regional and tertiary level health facilities their entry point into the health system, as shown by solid black lines in Figure 1 [5,6,7]. Under normal circumstances (e.g., noncritical situations), such an approach can be considered as an inappropriate use of health services. It sometimes leads to congestion at higher-level health facilities and unnecessary costs for both the patients and the health system [2,6,9,13]. In Ghana and other developing nations, studies show that the majority of clients seen at the outpatient units in secondary facilities could be treated appropriately at primary health care centres [5,7,8]. A consequence of this may be inappropriate use and potentially wasteful or even dangerously neglected non-use of appropriate health services [14]. The situation begs for research-based answers and strategies to address the gaps in the policy. 

According to Ghana’s Ministry of Health, the majority of the population/patients disregard the referral policy due to either ignorance or poor perceptions of the policy [7]. Some studies assert that low knowledge and the inability of many to distinguish between the functional differences of various health facilities weakens the referral system [11,12]. Therefore, factors outside the direct jurisdiction of the supply side of the health system do affect the functioning of the policy. In fact, the responsibility to adhere to the referral policy also rests significantly on the patients and their informal caregivers, as the initial part of Figure 1 indicates. The present study argues that attention to some demand-side factors—along with provider characteristics—will enrich understanding for a more efficient implementation and monitoring of the referral policy. Ultimately, if proper utilisation were to be frequent, this would assist the efficiency of the system and make its resources more useful for all concerned.

Studies indicate that the social environment of every setting shapes health outcomes and health interventions substantially [15,16,17]. Notably, the social relationships of patients and even those of the health personnel can affect health interventions [16]. This study advances the notion that social capital (SC) can offer an alternative approach to understanding the state of and challenges facing referral policy in Ghana. Social capital refers to the resources—information and emotional or instrumental support (e.g., money)—embedded in actual or potential social relationships [18,19,20,21]. It comprises two significant aspects, structural and cognitive SC components [15]. The structural aspect encapsulates the nature, intensity, and degree of closeness among members of a given relationship. Structural social capital has been conceptualised as having two primary forms, horizontal and vertical. Horizontal SC relates to bonding SC. Bonding SC consists of relationships between individuals and groups of equal or near-equal status regarding, for instance, age, power and resources [15]. Some of the most common examples include families, kin members, and close friends. Vertical SC, which is also referred to as ‘linking SC’, relates to hierarchical or unequal relationships between individuals with differences in power, resources and social status [15]. A typical example is a relationship between people and the leaders of communities or public and private institutions. Relationships with civil servants and professionals such as health personnel and educators can be considered as forms of linking social capital in deprived communities [15]. There is also bridging SC. It constitutes relationships across class, ethnicity, religion, and weak friendships (for example, a friend of a friend) [15]. All these horizontal and vertical relationships and the resources embedded in them embody structural SC [15]. The cognitive component of SC consists of the abstract elements of relationships, such as interpersonal trust, a sense of harmony and shared norms and reciprocity [15,22]. Many argue that social relationships are especially influential because of these cognitive elements [23,24]. 

The well-known theory of lay referral stipulates that the social relations of a sick person influence the decision to enter the health system and the choices made during and after using health services [25,26,27]. This is because illness and health have strong socio-cultural connotations [27,28]. Studies indicate that the majority of hospital users in Africa and Ghana are in fact ‘self-referrals’. Families, communities and peer-induced factors sometimes encourage people to skip or bypass lower level health services in favour of higher-level ones at the onset of a health problem [29,30]. Research in many parts of Ghana have consistently registered that reliance on both close and distant social networks—including cognitive elements such as trust—lead to adoption and conflation of different forms of health practice and options without recourse to the referral system [31,32]. Developments in other sub-Saharan African countries [13,33] and other parts of the globe, especially low- and middle-income countries [34], have also shown similar patterns. However, none of the studies cited has examined the state of the referral policy from an explicit SC standpoint. This study examines the nuances of how elements of social capital influence knowledge of, and adherence to, the referral policy amidst the efforts of health personnel and effects of other influential factors. By specifically addressing the relationship between SC and the referral policy, the present study uniquely provides critical insights into how one of the most prominent contemporary theories in social health research can be exploited to strengthen health service delivery in Ghana.

## 2. Methods

### 2.1. Study Design

The data for this paper emerged from an ongoing study whose primary objective is to tease out the extent of influence of different forms of SC on healthcare access and health literacy among rural and urban people in the Ashanti Region in Ghana. As part of the original study, the Committee on Human Research Publication, and Ethics (CHRPE) of the School of Medical Sciences, Kwame Nkrumah University of Science and Technology and Okomfo Anokye Teaching Hospital, Kumasi, Ghana, (CHRPE/AP/345/15) provided in-country ethical approval. The Regional Health Directorate gave written permission for the study. Participants were given adequate information about the aim of the research and their expected contribution before being enrolled in the study. 

The study used a qualitative deductive approach to generate data on how different social capital proxies affect knowledge and enforcement of the referral policy. It employed individual in-depth interviews and focus group discussions using a semi-structured interview guide [35], to give balanced and emic insights into the research problem. The study triangulated these the two data collection methods to ensure the credibility of the research process and the findings [35,36].

### 2.2. Data Collection and Sampling 

The study adopted a purposive sampling technique to elicit data from participants aged 18 years and older. The purposive sampling technique helped to capture the diversity of the study population in terms of age, gender, the location of residence, religion, and ethnic composition. Seventy-nine primary participants consisting of 28 and 51 people from eight rural communities and 36 urban communities, respectively, took part in the individual in-depth interviews and the focus group discussions. Six focus group interview sessions were conducted, with groups consisting of five persons on average. Each group comprised those who had participated in the individual interviews and new participants. The study also included 16 key informants, eight community leaders and eight health officers, who added contextual and professional perspectives to the discussions. The research team identified a gatekeeper in each of the communities. These people helped in establishing contacts with local political and traditional leaders. The purposive technique was used in health institutions to select personnel with different specialities (medical doctors, nurses, health promotion officers, and pharmacists). The data collection involved a one-time face-to-face interview with the participants. The activity lasted from June to October of 2015. Table 1 shows some of the socio-demographic characteristics of the primary participants. 

The first author, with the help of two research assistants, conducted all the interviews. Each interview session was audio-recorded with the permission of the participants. However, a note-taker was always present to record key points that emerged from the discussions. The local dialect, ‘Twi’, was used to conduct interviews with primary participants and community leaders. Interviews with health personnel were carried out using the English language. The interviews usually lasted from 35 to 60 min. The majority of interviews took place at the homes or workplaces of the participants. 

The introductory part of the interview sessions dealt with participants’ views and knowledge of, and experiences with, the referral system and the challenges they experienced in adhering to the process. Further to that, the interviews necessitated participants to report on how they involved their social relations in decisions and choices regarding when and where they sought medical help. The discussions with key informants concerned how different social connections of the sick affected awareness of, and adherence to, the referral process. The research team transcribed all of the interviews. An independent local language expert assessed the validity of the transcripts. 

### 2.3. Data Analysis

The analyses commenced with a thorough reading of the transcripts to become acquainted with the data and to seek explanations for difficult expressions and unfamiliar statements. The main analyses were conducted using a deductive and narrative approach to thematic construction. The concept of social capital guided the process [35]. The proxies of social capital—bonding, bridging, linking and cognitive forms—were used to guide the coding and to create categories (recurrent topics and experiences) and subcategories of the data. In the process, similar codes and subcategories were grouped under specific social capital proxies to form a theme. The codes and subcategories were constantly compared and (re)arranged to ensure that common codes were classified under the right theme. The results were interpreted through argumentative and narrative reasoning to demonstrate how relevant aspects of social capital alter the state of the referral policy. One social health scholar and a sociologist helped to validate the representations and analyses presented in this paper. 

## 3. Findings

The knowledge and willingness of participants to adhere to the referral policy were limited. This was typified by the predilection of many people to keep their ailments from further investigation—an activity which is fundamental to the referral process:

…Some patients and their relatives occasionally resist referral. …Usually, the patients would say they are scared to go to a higher-level facility. …When you refer a patient to Komfo Anokye (tertiary hospital), they may eventually go, but first, they would insist that they want to stay here for the same treatment. …They consider that going to Komfo Anokye means that one has a serious ailment and is on the verge of dying. …We have to spend time to convince them of the fact that the receiving facility is better equipped to handle their situation. …Many do not understand the referral processes (Health officer 3, urban)

As regards the substantive matter, the findings supported the conjecture that social capital contributes to the public’s knowledge of and adherence to the referral system. Elements of SC underscored how other factors such as financial difficulties, transport, and the availability of different forms of health services affected compliance with the referral policy.

### 3.1. Bonding Social Capital Determined Choice of Health Facilities

Many participants relied particularly on their close relatives and friends for all forms of support especially in times of ill health. Such relations played a crucial role in the decisions, and choices about health and healthcare as accounts of some participants revealed:

I often ask for help on where to seek medical help. …I mostly discuss it with my friends. …when I am not feeling well, I just tell them about how I am feeling. Usually, one of them will either recommend drugs for me or direct me to a good health facility to seek medical care (Ibrah, 50 years, urban)

The advice that participants received from the usual circles determined whether a person would make use of a health facility. The consultations further altered attitudes and decisions about compliance with the referral policy at the outset of a health problem. More often than not, participants adopted the referral system appropriately when they had the right information, or at least knowledge of the system. Wrong information or misinformation often resulted in misuse of the policy from the outset. For instance, in the case below, the participant eventually used a secondary health facility as her first-choice facility primarily due to incorrect information through bonding SC:

…When my child was sick, a lot of that happened. …Many people suggested different health centres to me. …Some asked me to go to Suntreso hospital [District hospital] …and Komfo Anokye [teaching hospital] …They went like …’This place is right, the new doctor is very good’. …Eventually, I took her to the Ejisu (District Hospital) for treatment. …It worked for me (Naa, 40 years, rural)

Therefore, aside from confiding in families and friends for initial support and information on care seeking, there was always the hidden, yet, imminent desire to source alternative information on places to receive ‘quality’ care.

### 3.2. Linking Social Capital Persuaded People to Abandon the Referral Policy

Linking SC, particularly association with health professionals/institutions, appeared to be either a deterrent or encouragement for the efficient use of health services concerning the referral policy. Indeed, associations with health workers proved many participants with first-hand information about essential health interventions and policies. Notwithstanding this, some health workers sometimes advised and even aided (potential) patients to access tertiary level facilities instead of primary or even secondary level facilities for initial assessment of their health problems. In fact, some participants were accustomed to such practices even to the extent of advocating more of such relationships:

…Every person needs to have a doctor or nurse as a friend so that in times of sickness, you can contact them for help. …I have a nurse as a friend …and I talk to her about my health. …She sometimes arranges for me to see specialist doctors at Komfo Anokye (tertiary hospital) (Akua, 42 years, urban)

In the study, reliance on such unequal relationships was indeed common as participants in both rural and urban localities echoed the same refrain. The availability of such social networks apparently inhibited understanding of or adherence to the policy and encouraged a dismissal of the referral process sometimes even at the request of a health professional. 

### 3.3. Bridging Social Capital Protected Against Misuse of the Referral Policy 

In most cases, issues of financing underscored decisions to either ignore or adhere to the referral policy. Financial difficulties impeded opportunities to gain further understanding and engagement with the referral system. However, there were indications that a high stock of bridging SC hampered the adverse implications of financial challenges regarding compliance with the referral system by enabling access to finances to obtain the needed health services:

My child fell sick for a while after I gave birth. I was referred from Foase (secondary hospital) to KATH (tertiary hospital). …I decided to go, but I realised that I did not have enough money. …I used the little money on me to get blood tonics for the child, but her condition did not improve after a while. …Later, one of my friends led me to acquire a loan from someone she knew. …I was then able to take him to the KATH (Akua, 35 years, rural)

Many participants—particularly rural residents—reported of difficulty in honouring referrals due to lack of transportation. In such instances, neighbours often carried the sick person physically to the closest health facility.

…When someone is referred to Foase (District hospital), and there is no car immediately, the men in the community organise themselves to carry the person to the hospital if he or she is not able to find a car after waiting for a while. …If the men do not help, then the patient would have to wait until the next day. …However, in all the cases that I have witnessed here, people were always ready to help once the patients and their families expressed the willingness to honour the referral (Health officer 2, rural)

The influence of structural SC on the referral policy in this regard moved beyond bonding SC to bridging SC. 

### 3.4. Social Norms and Adherence to the Referral Policy

Aside from issues relating to financing and transportation, some local values placed on certain practices and beliefs shaped the choices people made about health services. The social acquaintances of participants sometimes reinvigorated or in some cases enforced conformity to such practices. Some health workers considered such practices to be one of the major determinants of adherence to and even awareness of the policy. In some instances, strict reliance on such practices led to the loss of life:

Some time ago, a woman in labour had complications at the CHPS compound at Sanyeneso No.3. …The attendant handled the case, but she encountered some complications. …The patient had a retained placenta. …The nurse immediately asked those who brought the woman to the clinic to take her to another hospital. …She gave them a referral note as well. …Instead of taking her to the hospital, they took the patient home and asked her to blow air into a bottle to force her placenta out. …There is a perception that blowing air forcefully into a bottle could pressure out the placenta in such situations. She bled profusely and died eventually (Health officer 2, rural).

Moreover, the meaning ascribed to certain diseases as regards their aetiology and effects shaped how people engaged with the health system. For example, some participants first consulted a religious healer whenever they considered an ailment as spiritual. Sometimes, such decisions rested with the family and friends of the sick person, irrespective of the recommendations of a health professional:

The people know we do not treat such cases (diseases attributed to spiritual causes) here, so they take to the fetish priest for (spiritual) treatment. …Sometimes, the fetish priest consults us for assistance when he feels that the patient could use orthodox medical care whiles he continues with his spiritual work. …He sometimes asks us to treat his clients with wounds and severe cuts at his premises. …The relatives of the sick only consider our services if the fetish priest recommends it (Health officer 1, rural)

Therefore, the belief in alternative practices had an immense influence on the use of the formal and orthodox health system. To some extent, the social acquaintances of the sick orchestrated a cordial relationship between the two foremost health practices (conventional and unconventional medical practices), regarding the referral policy.

### 3.5. Trust Influenced Adherence to the Referral Policy

Some participants questioned the quality and cost-effectiveness of health services offered at some health facilities. Therefore, they used only the services recommended by their confidants—including their bonding and even bridging SC:

I always used to attend antenatal and postnatal services at Ejisu hospital, but now I use the clinic in the community (CHPS compound). …This is the only time I am using this facility (CHPS compound). ...This time, when I first realised that I was pregnant, I asked my sister-in-law who has been using the clinic here. …She told me the service is the same as that of Ejisu. …That was when I decided to visit this clinic (CHPS) (Naa, 40 years, rural).

Notwithstanding, the depth of trust in a relationship determined the extent of adherence to such recommendations. For instance, trusting relations inspired the decision to skip an available primary health facility for a secondary facility as this case shows:

Yes, I live here (Fori), but I go to Abuk (a place at the other end of the city) for healthcare. …I used to visit one clinic (a primary level facility) here, but I stopped. …Everyone in my house uses a facility at Abuk (secondary level facility), and none of them has ever complained about anything, so I decided to go there as well. …I felt that the facility at Abuk may be better than the one here (Victor, 32 years, urban).

However, the element of trust also appeared to limited the extent to which patients made use of the referral system. It emerged that some participants refrained from taking advice from even some close social ties because they considered them as lay persons in relation to health matters:

No, personally I do not share my health issues with other people except it is something that overwhelms me. I do not usually ask anybody about where to seek medical attention. …I only open up to discuss my health issues with people I consider as trustworthy and knowledgeable (Faust, 38 years, rural).

Hence, the element of trust either expanded or weakened conformity to the referral policy depending on the extent of the perceived competence of one’s social acquaintances.

## 4. Discussion

The findings highlight the essence of SC in the positive and negative functioning of the referral policy in the study context. As expected, the knowledge, ability, and willingness to adhere to the referral system were dependent not only on individuals and health workers but also their immediate and distant social relations. Moreover, cognitive forms of SC, including trust and social norms, moulded the extent of the influence of other forms of SC on the policy.

Ordinarily, the decision to seek care for common and recognised illnesses (such as malaria) in Ghana tends to be tardy [37]. Factors such as increasing user fees, expensive transport fares, negative past experiences in accessing health service, and the economic and social cost of forgoing other productive duties, tempt many to seek alternative sources of care, perhaps with the help of their families and friends, or even to delay or forgo treatment altogether [37,38]. In addition, people sometimes activate the resources (such as information sources and financial support) embedded in their social networks when their health problems are at advanced stages [37]. Late recourse to such resources sometimes leads to extemporaneous decisions about when and where to seek medical attention. In part, this explains why some participants ended up dismissing or bypassing the referral policy when they finally decided to engage with the formal health system. Previous studies support the observations in the present study. A related study in the Komenda–Edina–Eguafo–Abrem District in Ghana found that even the idea of being referred was a major deciding factor in the choice of health facilities at the onset of a disease [39]. Such attitudes help to explain why some participants consulted their relatives in choosing health centres. One can also speculate that the consultations that people make before seeking care were a means to avoid facilities to which they were likely to be referred.

The findings indicated that cognitive SC (trust and social norms) led to a disregard of the prerequisites set forth by the formal health system. Indeed, studies substantiate the conclusion that the social meaning attached to many health conditions paves the way for people to trust the opinion of their friends, relatives and work colleagues [28], apparently over that of the health service system. This adds to why some participants often presented dilemmas about healthcare to their close social ties instead of health professionals. Furthermore, recent studies support the finding that cultural precepts such as customs and belief systems sometimes deterred people from taking up the necessary health services correctly [30]. Gupta, Aborigo [40] argue that the value placed on such sociocultural precepts in Ghana encourage people to go the ‘extra mile’ to comply with these suggestions even against medical advice or personal inclinations, just as some health personnel in this study insinuated. Nonetheless, the influence of cognitive elements on referral behaviour fluctuated. This fluctuation may explain why some participants demonstrated trust in health professionals and institutions as opposed to their relatives and friends for health advice. To some extent, this finding buttresses the assertion that cognitive social resources such as trust are fluid [15,41].

Furthermore, the findings indicated that hierarchical relations with people who are knowledgeable or abreast of the referral system (mainly health personnel) did not necessarily deter the propensity to disregard the referral policy. Some health workers assisted clients to access high order services without going through the established process. This points to the downside of SC (linking SC, in this respect) [15] for the health system. The evidence is thus contrary to the assertion that individuals who are knowledgeable about health matters positively influence the actions of their acquaintances regarding choices and decisions in using healthcare [42]. In the study context, the inconsistency was partly due to numerous constraints in accessing healthcare [2,5]. On a regular basis, people have to manoeuvre their way out of expensive care [2]. The difficulties in accessing health care explain the informal reliance on health personnel for health services, which sometimes encouraged disregard for the referral policy. To some extent, the poor state of the referral system in practice, as the Ghana Health Service (GHS) acknowledges, underscores its dismissal by the public [5,6,7]. It is thus perhaps not unreasonable to argue that the state of the policy discourages even health personnel from enforcing the systematic use of the health system. One could also claim that the health workers themselves may be doing little to foster the efficiency of the policy and public trust in it. Apparently, such weak efforts underline the ‘ignorance’ [7] of the public regarding the policy. Nevertheless, the negative behaviours of participants regarding the adoption of the official referral pathway were similar to the findings from earlier works in Ghana [10,32,43]. Similar observations have also been made by studies in other sub-Saharan countries such as Uganda [11] and Nigeria [13]. 

Nonetheless, reliance on SC for support in times of ill health sometimes indirectly contributed to adherence to the referral process. Across sub-Saharan Africa, issues relating to finance or costs are the most likely reason why people ignore medical needs [31,33]. There is a provision for a financial safety net for health in Ghana in the National Health Insurance Scheme (NHIS). The NHIS is meant to ensure equitable access to healthcare [2,44]. However, the policy is currently a shadow of its formative period. Studies suggest that people now prefer to pay for healthcare by themselves as the NHIS offer a piteous service due to financial and administrative challenges [1,2]. The poor state of such welfare policies partly explicates the inability of some participants to adhere to formal referrals. In the light of such contextual challenges, social capital becomes something of a substitute for social safety net programmes such as the NHIS, which are supposed to address inequalities in health care access. The role of SC in healthcare financing is however not only prevalent in Ghana and similar LAMICs. Rostila [45] postulates from studies of European welfare regimes that “vulnerable citizens residing in these countries [countries with low social security] may have to rely on their social networks for different kinds of material and economic support in situations of personal or financial difficulties due to poor social safety nets”. The discrepancies in such social interventions emphasise why financial assistance through SC encourages people to honour referrals. Indeed, without the role of SC, many people would dismiss referrals without hesitation. From another perspective, the support through social capital to honour referrals can reduce wasteful healthcare expenditure, which arises when people attempt to take up (cheaper) alternative services in a bid to avoid perceived costly expenses by adhering to referrals. In the long run, such ad hoc expenses may only have minimal benefits, as Bakare [33] has observed in Nigeria. 

The initial stage of health service delivery in Ghana recognises the role of social relations in the uptake of healthcare, in the form of home care and decision-making (Figure 1). However, the role significantly subsides after entry into the formal health system, as official documents and procedures (see [7]) fail to recognise the input of social relations. In the light of the findings of this study, the referral system can be enhanced by formally recognising or acknowledging the role of SC in two more stages. This study proposes that the first point of intervention (Point A in Figure 1) should be after engagement with the primary health care facilities. The next point should follow the secondary level of care (Point B) if the need arises. Considering the precarious nature of Ghana’s health system [2], it will be prudent to make room for the role of social capital binding. The referral forms could make room for relatives by tasking them to relay information (on successful or otherwise completion of the referral/transfer process). Formal recognition of the relatives could curb the tendency of families and friends to dissuade the sick from either taking up or completing the referral process as the findings indicated.

The results of this study should nevertheless be interpreted in the light of some limitations. While care was taken to transcribe the interviews from the local dialect into English for analysis, an entirely accurate translation of the data cannot be assured due to potential human errors. However, repeated validation by the research team coupled with the assistance of the language expert assured the credibility of the data. Also, not all the forms of SC demonstrated a substantial effect on the referral policy. To this end, some care is needed in interpreting the findings and especially for extrapolating them to other contexts, including even those in Ghana, as the study only focused on the Ashanti Region. Future studies could examine the research question by a mixed methods research approach and possibly a multi-region project. Such a design may expand the knowledge base on specific dimensions of social capital that influence the referral policy. 

## 5. Conclusions

The study sought to examine the relationships between SC and the referral policy in the Ashanti Region of Ghana. The findings revealed that different aspects of SC have both positive and negative influences on the policy. Some aspects of SC have a direct bearing on the effectiveness of the referral process through persuasion and indirectly through factors such as finance and transportation. Such influences promote awareness and even understanding of the policy. Notwithstanding this, the effect of SC on the referral policy is tied to the ‘resourcefulness’ (such as financial power and knowledge) and ‘trustworthiness’ of an individual’s social networks. Moreover, the potential of SC to enhance or improve the effectiveness of the referral policy is not unique to the study context, as the findings correlate with previous works in both developed and low- and middle-income countries. Regardless of some of its potentially adverse effects, a careful approach to incorporating SC into the referral policy has the potential to mitigate some of its longstanding challenges significantly. 

## Figures and Tables

**Table 1:** Characteristics of Participants in the Study. Source: Authors’ Construct.

Characteristic	Urban (*n* = 28)		Rural (*n* =51)		Overall (*n* = 79)	
	Frequency	Percentage	Frequency	Percentage	Frequency	Percentage
Sex						
Male	13	46.43	24	47.06	37	46.84
Female	15	53.57	27	52.94	42	53.16
Age						
18–24	5	17.85	7	13.72	12	15.19
25–34	8	28.57	14	27.45	22	27.85
35–44	8	28.57	12	23.53	20	25.32
45–59	3	10.71	12	23.53	15	18.99
60+	4	14.29	6	11.76	10	12.65
Educational Attainment						
Never been to school	4	14.29	12	23.53	16	20.25
Primary school	7	25	12	23.53	19	24.05
Junior High Sch.	10	35.71	14	27.45	24	30.38
Senior High Sch.	4	14.29	9	17.65	13	16.46
Tertiary Level	3	10.71	4	7.84	7	8.86
Total	28	100	51	100	79	100
